# A Text-Book of Physical Chemistry

**Published:** 1900-06

**Authors:** 


### Reviews of Books.

**A Text-Book of Physical Chemistry.** By Dr. R. A. LEHFELDT. Pp. viii., 308. London: Edward Arnold. [1899.]

The importance of physical chemistry has only been recognised during the last decade, and Professor Lehfeldt is to be congratulated on being the first to write an English book on the more modern aspects of the science; and it is a well-written and readable treatise, " by no means written to suit any examination." The modern theory of solution is clearly stated; unless the exact mechanism of solution is known, it is impossible to form a sound judgment on physiological processes, or an explanation of the phenomena of absorption and secretion; but in spite of this it comes into no medical curriculum and is quite unknown to the average medical student. Chapter ii., on Physical Constants, contains a brief and accurate account of S. Young's work on van der Waal's theorem. The omission here of specific-heats and of Dulong and Petit's classical work is noticeable; the subject is dismissed in another part of the book as being an ill-defined quantity, but its great value to the atomic theory of matter would alone have warranted a better notice. When dealing with the subject of polarised light, it might have been more clearly brought out that such investigations have led to one of the most fascinating developments of chemistry in recent times, the tri-dimensional conception of the arrangement of the atoms in the molecule. The greater part of the work is devoted to the extremely important principles of thermodynamics and their application to chemical problems, and is written in such a manner as to be particularly useful to the more advanced student.

